# No Clinical Benefit to Treating Male Urinary Tract Infection Longer Than Seven Days: An Outpatient Database Study

**DOI:** 10.1093/ofid/ofz216

**Published:** 2019-05-06

**Authors:** George J Germanos, Barbara W Trautner, Roger J Zoorob, Jason L Salemi, Dimitri Drekonja, Kalpana Gupta, Larissa Grigoryan

**Affiliations:** 1 Department of Family and Community Medicine, Baylor College of Medicine, Houston, Texas; 2 Section of Health Services Research, Departments of Medicine and Surgery, Baylor College of Medicine, Houston, Texas; 3 Houston VA Center for Innovations in Quality, Effectiveness and Safety (IQuESt), Michael E. DeBakey Veterans Affairs Medical Center, Texas; 4 Department of Medicine, Infectious Diseases, Minneapolis Veterans Affairs Health Care System, Minnesota; 5 Section of Infectious Diseases, Department of Medicine, Boston Veterans Affairs Healthcare System and Boston University School of Medicine, Massachusetts

**Keywords:** antibacterial agents, antibiotics, resistance, stewardship, urinary tract infections

## Abstract

**Background:**

The optimal approach for treating outpatient male urinary tract infections (UTIs) is unclear. We studied the current management of male UTI in private outpatient clinics, and we evaluated antibiotic choice, treatment duration, and the outcome of recurrence of UTI.

**Methods:**

Visits for all male patients 18 years of age and older during 2011–2015 with *International Classification of Diseases, Ninth Revision, Clinical Modification* codes for UTI or associated symptoms were extracted from the EPIC Clarity Database of 2 family medicine, 2 urology, and 1 internal medicine clinics. For eligible visits in which an antibiotic was prescribed, we extracted data on the antibiotic used, treatment duration, recurrent UTI episodes, and patient medical and surgical history.

**Results:**

A total of 637 visits were included for 573 unique patients (mean age 53.7 [±16.7 years]). Fluoroquinolones were the most commonly prescribed antibiotics (69.7%), followed by trimethoprim-sulfamethoxazole (21.2%), nitrofurantoin (5.3%), and beta-lactams (3.8%). Antibiotic choice was not associated with UTI recurrence. In the overall cohort, longer treatment duration was not significantly associated with UTI recurrence (odds ratio [OR] = 1.95; 95% confidence interval [CI], 0.91–4.21). Longer treatment was associated with increased recurrence after excluding men with urologic abnormalities, immunocompromising conditions, prostatitis, pyelonephritis, nephrolithiasis, and benign prostatic hyperplasia (OR = 2.62; 95% CI, 1.04–6.61).

**Conclusions:**

Our study adds evidence that men with UTI without evidence of complicating conditions do not need to be treated for longer than 7 days. Shorter duration of treatment was not associated with increased risk of recurrence. Shorter treatment durations for many infections, including UTI, are becoming more attractive to reduce the risk of resistance, adverse events, and costs.

Urinary tract infections (UTIs) are the most common bacterial infections in the United States and the most frequent cause of bacteremia in men [1–4]. Incidence of UTI in the community is 0.9 to 2.4 cases per 1000 men below the age of 55 years; however, incidence reaches 7.7 per 1000 in those 85 years and older, approaching rates seen in women of the same age group [3, 5, 6]. Increasing rates of antibiotic-resistant infections, including those of the urinary tract, due in part to medically unnecessary prescriptions, have increased the need for antimicrobial stewardship when treating UTI in clinical practice [7–9].

Antimicrobial stewardship strategies encourage using narrow-spectrum antibiotics for the shortest duration required for clinical recovery. However, in a recent review on management of UTI in older men, recommendations regarding the optimal duration of treatment were hampered due to the lack of clinical trials comparing the efficacy of different regimens [3]. Current clinical recommendations regarding the management of UTI in men, as previously published, are shown in Figure 1 [3]. Most previous research assessing duration of antibiotic therapy for male UTI consisted predominantly of patients with complicated infections, including the presence of fever [10, 11] or catheter use [12, 13]. For example, a 2016 study by Mospan and Wargo [13], which reported a 5-day course of fluoroquinolones to be as effective as a 10-day treatment duration, excluded men with uncomplicated UTI entirely. This finding suggests that a 5-day treatment course is also likely to be adequate for uncomplicated UTI. Similarly, a large observational study at the Veterans Affairs health system found that men treated for 7 days or less have similar rates of UTI recurrence as those treated for longer duration [12]; however, the study included mainly older men with UTI and did not include information on the presence of fever or catheterization. A 2003 Scandinavian trial found similar outcomes between 2 and 4 weeks of antimicrobial therapy for men with febrile UTIs [10]. In contrast, a more recent study by van Nieuwkoop et al [11] found a subgroup of men with febrile UTI treated with 7-day regimens to have inferior clinical cure rates than those treated with 14 days of antibiotics. However, these 2 trials included only febrile UTI suggestive of pyelonephritis or prostatitis [10, 14]. We currently lack robust evidence guiding treatment duration for UTI in men in the outpatient setting [10, 14]. Thus, we aimed to address this gap in knowledge by studying the relationship between treatment duration and UTI recurrence in outpatient male UTI with and without complicating factors. Predictors of antibiotic choice and treatment duration, as well as the effect of treatment duration on recurrence of UTI, were assessed.

**Figure 1.**
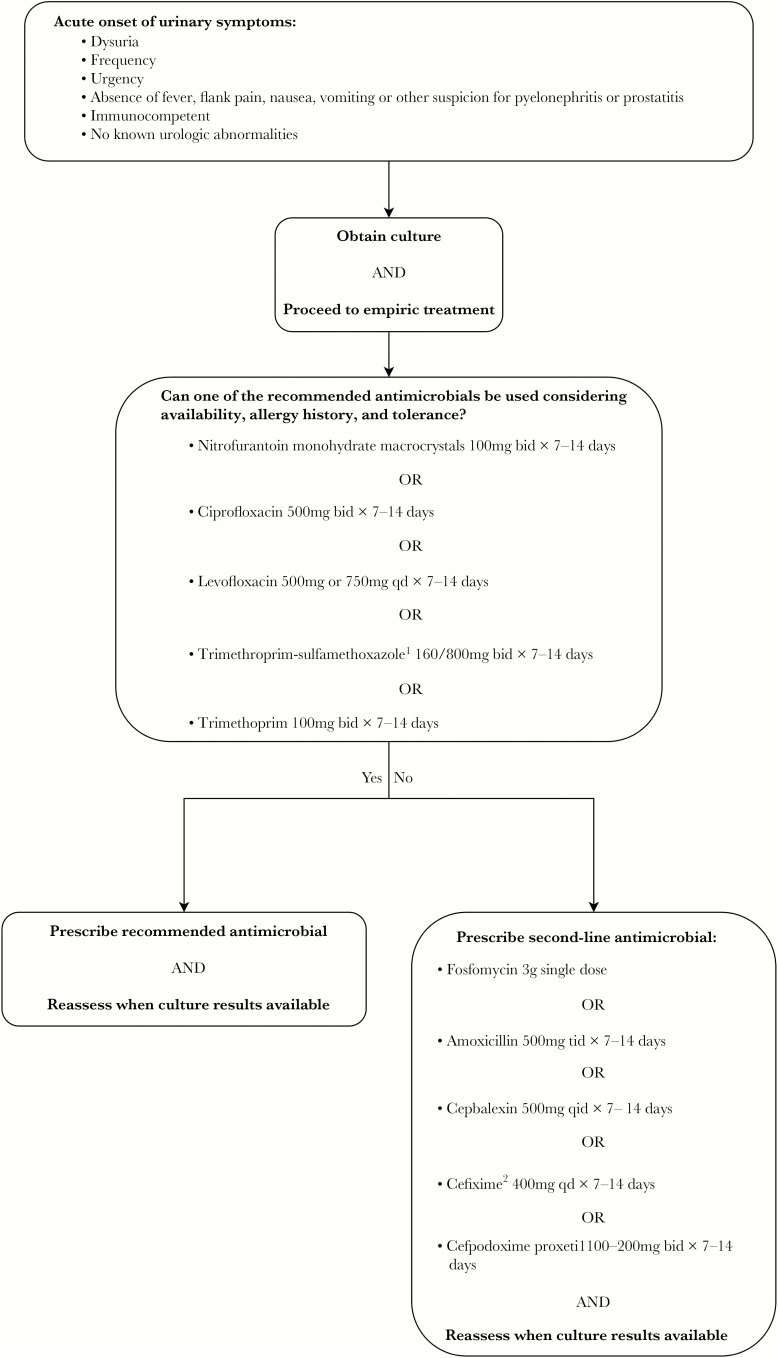
Approach to choosing empirical antibiotic treatment for male urinary tract infections. ^1^Avoid if resistance prevalence is known to exceed 20% or if used to treat urinary tract infection in previous 3 months. ^2^For resistant organisms. Adapted from Schaeffer AJ and Nicolle LE. Clinical practice. Urinary tract infections in older men. *N Engl J Med*. 2016;374:562.

## METHODS

### Setting and Study Population

This retrospective cohort study included patients from 3 different types of outpatient specialty clinics, including 2 family medicine, 1 general internal medicine, and 2 urology private practices. Deidentified records from an electronic health record (EHR) system (EPIC Clarity Database) were used to identify all men 18 years of age and older with any of 6 *International Classification of Diseases, Ninth Revision, Clinical Modification* (ICD-9-CM) diagnosis codes for UTI or lower urinary tract symptoms documented during an office visit between January 1, 2011 and September 30, 2015. These codes included 595.0 (acute cystitis), 595.9 (unspecified cystitis), 599.0 (UTI), 788.1 (dysuria), 788.63 (urgency of urination), and 788.41 (frequency of urination). In addition to a UTI-related diagnosis, patients must have also been prescribed a UTI-relevant antibiotic during the same visit. Urinary tract infection-relevant antibiotics included fluoroquinolones, nitrofurantoin, trimethoprim alone or in combination with sulfamethoxazole, beta-lactams, aminoglycosides, and macrolides. For each eligible visit, we extracted variables pertaining to the patient’s age, race, date of office visit, presence of fever, comorbidities, antibiotic prescribed, and duration prescribed, history of urogenital procedures or malignancy, anatomic abnormalities, as well as history of diabetes, benign prostatic hyperplasia (BPH), nephrolithiasis, and prostatitis. We also extracted data to determine a patient’s immune status, including whether the patient was being treated with biologics, undergoing chemotherapy, or prescribed steroids. For visits with recurrent UTI, we determined whether the uropathogen in the index visit was susceptible to the empiric therapy prescribed.

**Figure 2.**
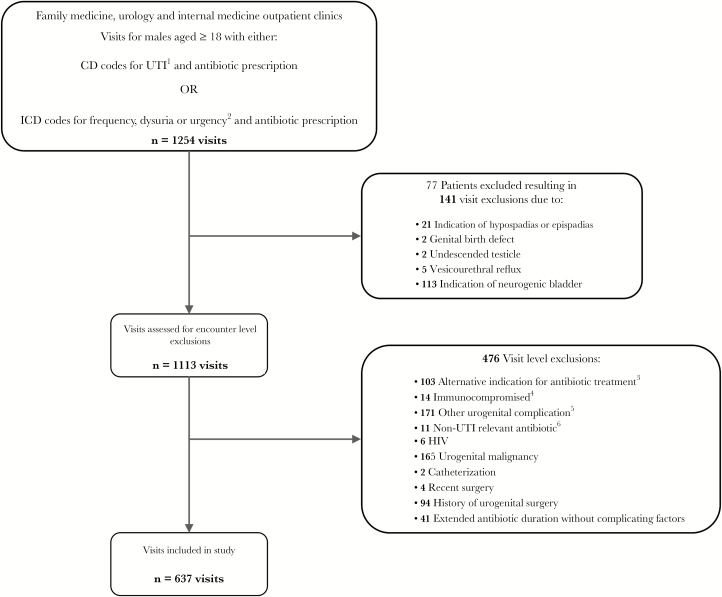
Selection process for urinary tract infection (UTI) visits. ^1^*International Classification of Diseases* (ICD-9) codes 595.0 acute cystitis, 595.9 cystitis unspecified, and 599.0 UTI site not specified. ^2^ICD-9 codes 788.1 dysuria, 788.63 urgency of urination, and 799.41 frequency of urination. ^3^Six miscellaneous infections, 4 pneumonia, 66 gastrointestinal, 14 mucosal, 8 sinusitis, 3 pharyngitis, 2 otitis, and 1 acne. ^4^Seven visits had active chemotherapy and 7 had active steroid therapy. ^5^Twenty-four visits had code for epididymitis/orchitis, 16 for other disorder of bladder, 17 other disorder of urethra, 103 urethral stricture, 9 unspecified disorder of kidney and urethra, and 8 syphilis. ^6^Four visits were prescribed azithromycin, 1 polymyxin B, 2 ceftriaxone, 1 clarithromycin, and 3 doxycycline.

Our process for applying patient-level and visit-level exclusion criteria to select eligible outpatient visits for inclusion in our analysis is described in Figure 2. We excluded all visits for patients with any history of anatomic abnormality including hypospadias, epispadias, other genital birth defects, vesicourethral reflux, or neurogenic bladder. In addition, visits with alternative indications for antibiotic treatment, non-UTI-relevant antibiotic prescribed, urogenital malignancy, other urogenital complications, catheterization, or indication of compromised immune status such as human immunodeficiency virus or active chemotherapy were excluded. All visits occurring within 30 days of recent surgery or after record of any major urogenital surgery were also excluded. Finally, visits with extended antibiotic duration of over 14 days without indication of complicating factors, defined as presence of pyelonephritis, nephrolithiasis, or prostatitis, were excluded.

### Outcome Variables

All UTI-related visits were classified as an index case, an early recurrence, or a late recurrence. Index cases were defined as the first visit with a defined UTI (clinical encounter with an associated ICD-9 code for UTI or symptoms, plus a prescription of UTI-relevant antibiotic) during the study period. Early recurrence was defined as a subsequent visit between 6 and 29 days after the index case with an ICD-9 code for UTI or symptoms plus a new prescription for antibiotic course. Late recurrence was defined as a subsequent visit between 30 and 365 days after the index case with ICD-9 code for UTI or symptoms, plus a UTI-relevant antibiotic prescription. We excluded visits with a second prescription occurring within 5 days of the index visit because these likely represent treatment of index event resulting from an adverse effect, loss of initial prescription, or ineffectiveness of the original agent.

### Covariates

The study’s primary exposure was treatment duration, dichotomized as short (≤7 days) or long (>7 days) based on expert recommendation for treatment of UTI [3]. Visits were then classified as having or not having complicating factors, based on the presence of 1 or more visit-level diagnoses of the following: pyelonephritis (590.xx), prostatitis (601.xx), or nephrolithiasis (592.xx, 594.xx). A modified version of the Charlson Comorbidity Index (CCI) was used to measure comorbid disease status and was calculated using ICD-9-CM codes associated with the visit in question or any prior visit [15]. Diabetes was excluded from the calculation of original CCI because it was included in the analysis as a separate variable. Patients were classified as having diabetes (1) if the diagnosis code (250.xx) was listed in the visit or the active problem list or (2) by measured glycosylated hemoglobin (HbA1c) ≥6.5%. Presence of fever was assessed using the temperature recorded during the visit. We could obtain reliable data on recorded temperature only in the family medicine clinics (temperature was missing for >90% of all urology and internal medicine clinics); therefore, the analyses including fever are restricted to these family medicine clinics only. Patient age at time of visit and any history of BPH (600.xx) were also extracted from the EHR.

### Statistical Analysis

We used χ^2^, Fisher’s exact, and Mann-Whitney *U* tests to compare patient characteristics and treatment regimens between visits stratified by presence or absence of complicating factors. Crude (unadjusted) and logistic regression models were used to identify relevant predictors of antibiotic choice; we included in multivariable models those factors that had a *P* < .10 in univariate analyses. Multiple linear regression analysis was used to identify the predictors of treatment duration. Logistic regression analysis was also used to calculate odds ratios (OR) and 95% confidence intervals (CIs) representing the association between the treatment duration and UTI recurrence. Factors were considered significantly associated with recurrence using *P* < .05. Data were analyzed using SPSS (version 24; SPSS, Chicago, IL) for Windows. This study was reviewed by the Baylor College of Medicine Institutional Review Board and was not considered to be human subjects research.

## RESULTS

### Visit Characteristics

Our study included 637 visits among 573 unique men seen between January 1, 2011 and September 30, 2015 (Figure 2). Patients visiting these clinics were predominantly white with private insurance. Of the 637 included visits, 119 (18.7%) visits had an indication of 1 or more complicating factors: pyelonephritis, nephrolithiasis, or prostatitis. Table 1 shows patient characteristics, antibiotics prescribed, and duration of treatment stratified by the presence of 1 or more complicating factors. Men without indication of a complicating factor were more likely to have been treated for a shorter duration and more likely to have diabetes. Overall, fluoroquinolones were the most commonly prescribed antibiotic class for all visits seen (69.7% of all visits). Trimethoprim with or without sulfamethoxazole (TMP-SMX) was the next most commonly prescribed antibiotic, followed by nitrofurantoin. Beta-lactams were the least commonly prescribed antibiotic class (less than 5% of all visits).

**Table 1.** Comparison of Patient Characteristics, Antibiotic Choice, and Treatment Duration Per Visit, Stratified by the Presence of Complicating Factors

Characteristic	Visits Without Complicating Factors^a^ (n = 518)	Visits With Complicating Factors^a^ (n = 119)	*P* Value
Age, median (IQR), years	57 (40–67)	52 (40–64)	.10
Race, n/Total n (%)^b^			**.005**
White race	350/457 (76.6)	80/101 (79.2)	
Black race	84/457 (18.4)	9/101 (8.9)	
Other race^c^	23/457 (5.0)	12/101 (11.9)	
Department, n (%)			.06
Family medicine	276 (53.3)	49 (41.2)	
Urology	211 (40.7)	61 (51.3)	
General internal medicine	31 (6.0)	9 (7.6)	
Fever, n (%)^d^	9/273 (3.3)	3/49 (6.1)	.40
Diabetes mellitus, n (%)^e^	57 (11.0)	4 (3.4)	**.01**
Charlson comorbidity index, median (IQR)	0 (0–0)	0 (0–1)	.11
Benign prostatic hyperplasia, n (%)	152 (29.3)	45 (37.8)	.07
Antibiotic, n (%)			.85
Fluoroquinolones	362 (69.9)	82 (68.9)	
TMP-SMX^f^	111 (21.4)	24 (20.2)	
Nitrofurantoin	27 (5.2)	7 (5.9)	
β-lactams	18 (3.5)	6 (5.0)	
Treatment Duration, n (%)			**<.0001**
≤5 days	96 (18.6)	5 (4.2)	
>5 and ≤7 days	167 (32.3)	13 (10.9)	
>7 and ≤10 days	161 (31.1)	33 (27.7)	
>10 and ≤14 days	93 (18.0)	29 (24.4)	
>14 days^g^	0 (0)	39 (32.8)	
Treatment duration, median (IQR), days	7 (7–10)	14 (10–28)	**<.0001**

Abbreviations: IQR, interquartile range; TMP-SMX, trimethoprim-sulfamethoxazole; Statistically significant results are shown in bold lettering.

^a^Complicating factors defined as visits with indication of pyelonephritis, nephrolithiasis, or prostatitis.

^b^Data are missing for 79 patients.

^c^“Other” race category includes American Indian, Asian, Chinese, Filipino, Hispanic, Hawaiian, Other Asian, Other Pacific Islander, and other.

^d^Recorded temperature ≥100.3°F available for visits in family medicine department only.

^e^Presence of diabetes mellitus indicated by active problem list or HbA1c measurement ≥6.5%.

^f^ Includes 1 visit with trimethoprim alone.

^g^Patients without a diagnosis code for pyelonephritis, nephrolithiasis, or prostatitis and with a treatment duration over 14 days were excluded from study population.

### Predictors of Antibiotic Choice

Age, CCI, and department were statistically significant predictors of antibiotic choice. Men 55 years and older were prescribed fluoroquinolones less frequently than younger men (65.4% vs 74.6%, respectively; *P* = .01), but they were more likely to have been treated with nitrofurantoin (7.4% vs 3%, respectively; *P* = .01). Patients with higher CCI were more likely to have been treated with a beta-lactam, irrespective of age (OR = 1.43; 95% CI, 1.04–1.95). Patients seen in the urology department were also more likely to be treated with a beta-lactam (OR = 6.7; 95% CI, 1.9–23.4) and were less likely to be treated with TMP-SMX (OR = 0.54; 95% CI, 0.36–0.82). Complicating factors, race, diabetes, BPH, and fever were not associated with antibiotic choice.

### Predictors of Treatment Duration

The presence of any complicating factor (pyelonephritis, nephrolithiasis, or prostatitis), antibiotic class, and the departmental specialty (family medicine, internal medicine, or urology) were significant predictors of treatment duration in the multiple linear regression analysis. Visits with complicating factors were treated for longer duration than visits without complicating factors (β-coefficient 9.038; 95% CI, 7.735–10.341; *P* < .001) after adjusting for antibiotic class and departmental specialty. Nitrofurantoin use was associated with longer treatment (β-coefficient 2.718; 95% CI, 0.256–5.181; *P* = .031), whereas use of beta lactams was associated with shorter treatment duration (β-coefficient −3.076; 95% CI, −5.950 to 0.202; *P* = .036). Visits in the urology department were more likely to be treated with longer courses of antibiotics (β-coefficient 2.360; 95% CI, 1.287–3.434; *P* < .001).

### Detailed Review of Index and Recurrent Visits

Overall, 32 patients (5.6%) had any recurrence with 7 patients having early recurrence, 25 patients having late recurrence, and 1 patient having both early and late recurrence. For all patients with recurrent UTI, we performed a detailed review of the index and recurrent visits and extracted all urine culture results and antibiotic susceptibilities. All subsequent visits represented acute symptomatic UTI with a new antibiotic prescribed for UTI recurrence. The uropathogen in the index visit was susceptible to the empiric therapy prescribed except in 2 cases of TMP/SMX resistance. Thus, resistance to initial therapy could not be evaluated as a factor for predicting recurrence.

### Relationship Between Treatment Duration and Recurrence

**Figure 3.**
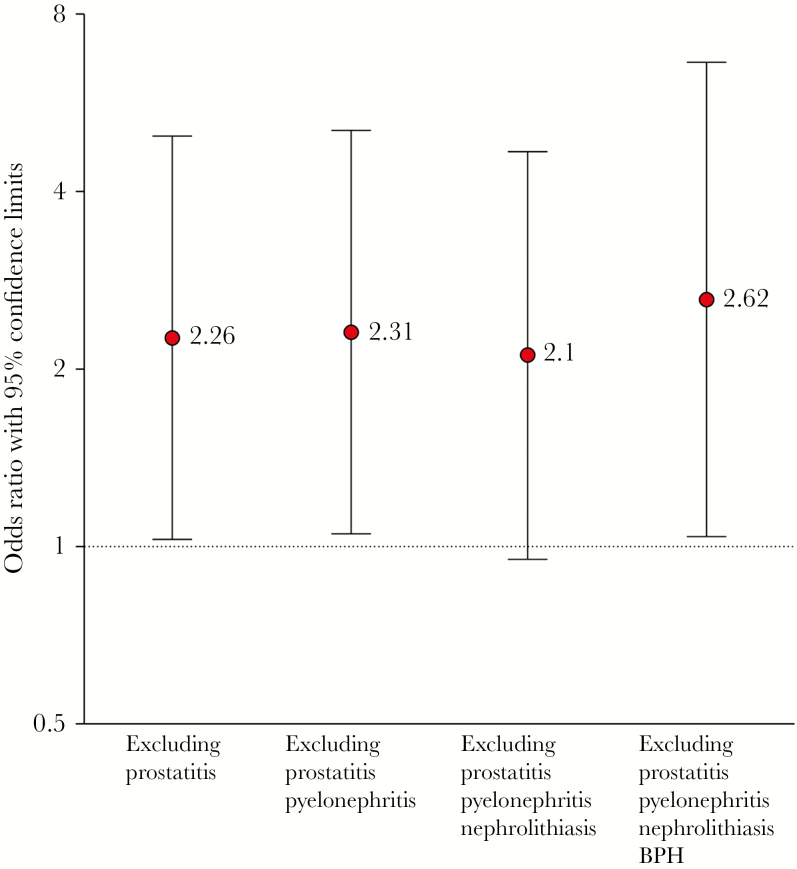
Risk of recurrence with longer antibiotic treatment in men with urinary tract infections without predisposing factors. Predisposing factors include anatomic abnormalities, history of urogenital malignancy or surgery, catheterization, or compromised immune status.

 In the overall cohort, longer treatment duration was not significantly associated with UTI recurrence (OR = 1.95; 95% CI, 0.91–4.21). Table 2 and Figure 3 show the results of separate logistic regression models assessing the association between treatment duration and UTI recurrence, for 4 subgroups of men with UTI, each subgroup defined by the complicating factors that were used as a basis for exclusion. In 3 of 4 subgroups, treatment duration longer than 7 days was associated with a higher risk of UTI recurrence. Relative to other subgroups, the magnitude of the association between treatment duration on UTI recurrence was the strongest (OR = 2.6; 95% CI, 1.04–6.61) in the subgroup that excluded BPH in addition to the complicating factors of prostatitis, pyelonephritis, and nephrolithiasis. To control each logistic regression model for potentially confounding variables, we included age, CCI index, presence of diabetes, department (urology versus family and internal medicine), race, and antibiotic type. None of these factors were significant and were therefore not included in the final model.

**Table 2.** Bivariate Analyses of Predictors of UTI Recurrence in the Overall Cohort and in Each Subgroup

Predictor	Overall Cohort	Visits Without a Diagnostic Code for Prostatitis	Visits Without a Diagnostic Code for Prostatitis or Pyelonephritis	Visits Without a Diagnostic Code for Prostatitis or Pyelonephritis or Nephrolithiasis	Visits Without a Diagnostic Code for Prostatitis or Pyelonephritis, Nephrolithiasis or BPH
	N = 573	N = 493	N = 488	N = 467	N = 331
Longer duration	1.95 (0.91–4.21)	**2.26 (1.03–4.96)** ^a^	**2.31 (1.05–5.08)**	2.11 (0.95–4.68)	**2.62 (1.04–6.61)**
Patient age (years)	1.01 (0.99–1.03)	1.01 (0.99–1.03)	1.01 (0.99–1.03)	1.01 (0.98–1.03)	1.01 (0.98–1.04)
Patient race					
White	Reference	Reference	Reference	Reference	Reference
Black	0.72 (0.24–2.10)	0.77 (0.26–2.29)	0.77 (0.26–2.28)	0.76 (0.25–2.26)	0.44 (0.10–1.94)
Other	0.41 (0.05–3.14)	0.52 (0.07–3.97)	0.58 (0.07–4.52)	0.60 (0.08–4.68)	0.54 (0.07–4.26)
Charlson comorbidity index	0.98 (0.65–1.49)	0.95 (0.62–1.46)	0.95 (0.62–1.46)	0.95 (0.62–1.46)	0.90 (0.51–1.59)
Diabetes	1.40 (0.47–4.16)	1.42 (0.48–4.27)	1.41 (0.47–4.21)	1.39 (0.46–4.19)	1.42 (0.40–5.08)
Department					
Family and Internal	Reference	Reference	Reference	Reference	Reference
Urology	0.79 (0.38–1.64)	0.82 (0.38–1.78)	0.81 (0.37–1.75)	0.78 (0.35–1.73)	1.02 (0.40–2.59)
Antibiotic type					
Beta lactams	Reference	Reference	Reference	Reference	Reference
Fluoroquinolones	0.51 (0.11–2.36)	1.16 (0.15–9.08)	1.17 (0.15–9.22)	1.17 (0.15–9.22)	0.73 (0.09–6.04)
TMP-SMX	0.40 (0.07–2.21)	0.76 (0.08–7.18)	0.76 (0.08–7.18)	0.76 (0.08–7.18)	0.30 (0.03–3.66)
Nitrofurantoin	1.08 (0.16–7.14)	2.16 (0.21–22.49)	2.16 (0.21–22.49)	2.16 (0.21–22.49)	2.14 (0.19–23.72)

Abbreviations: BPH, benign prostatic hyperplasia; TMP-SMX, trimethoprim-sulfamethoxazole; UTI, urinary tract infection.

^a^Statistically significant results are shown in bold lettering.

## DISCUSSION

Antibiotic duration is often driven by a desire to reduce the risk of recurrent infection. In this retrospective cohort study of outpatient men with UTI, we found that longer antibiotic treatment was associated with 2-fold increased odds of recurrent UTI after excluding men with urologic abnormalities, immunocompromising conditions, prostatitis, pyelonephritis, nephrolithiasis, and BPH. This lack of benefit in reducing recurrent UTI was independent of age, CCI index, presence of diabetes, department (urology versus family and internal medicine), patient race, presence of fever, and the type of antibiotic. Our findings are in agreement with the results of a recent observational study performed in the Veterans Affairs population showing that longer treatment (>7 days) for male UTI was not associated with a reduction in early or late recurrence [12]. The current study expands on previous findings by focusing on men without urological anatomical abnormalities or instrumentation (such as urinary catheters) and by performing subgroup analyses of visits with prostatitis, pyelonephritis, BPH, or nephrolithiasis and controlling for comorbidities. Thus, our data support prescribing 7 days or less of antibiotic therapy for male UTI over a range of clinical presentations and at different clinic settings, including primary care and subspecialty clinics.

An unexpected finding was the higher rate of recurrence with longer therapy in the subgroup with fewer complicating factors. Our findings are consistent with previous observational studies in other patient subgroups that demonstrated lack of clinical benefit with longer antibiotic duration. Longer course of antibiotics was associated with a higher recurrence within 30 days in a Dutch observational study including women with UTI and diabetes [16]. Treatment duration longer than 5 days was associated with an increased risk of early recurrence in our previous study including women with and without diabetes [17]. Mechanisms for alternation of gastrointestinal flora as a result of antibiotic use have been suggested [18], and gastrointestinal flora can in turn impact urogenital flora [19, 20]. It is unclear whether the findings in our study are related to underlying host factors, or whether longer treatment alters the microbiome and increases the risk for recurrence, perhaps more so in patients who are otherwise healthier and presumably less exposed to antibiotics.

The most frequently prescribed antibiotics in our study were fluoroquinolones, which continue to be used as a first-line treatment despite rising rates of resistance [21, 22], and recent US Food and Drug Administration warnings advising against their use for uncomplicated UTIs when alternative antibiotics can be prescribed [23, 24]. In our analysis, antibiotic choice was not associated with UTI recurrence, which may support use of nitrofurantoin or TMP-SMX as a first-line treatment in our patient population.

The results of our observational study must be interpreted with caution, particularly due to the possibility of confounding by indication. Longer courses of antibiotics could have been prescribed for sicker patients, thus confounding the relationship between treatment duration and recurrence. We tried to minimize this bias through rigorous eligibility criteria and by including a comorbidity burden indicator in our multivariable analyses. In addition, the data used in our study were collected for administrative and billing purposes and not for the purpose of research. As a result, visits analyzed may be subject to misclassification and not be indicative of a UTI, particularly visits identified by symptom codes rather than ICD codes. Our inclusion criteria required a UTI-relevant antibiotic to have been prescribed at the time of the visit, which we believe indicates provider suspicion of infection. To exclude visits in which antibiotics may have been prescribed for nonurinary infections, visits with any alternative indication of infection were excluded from our study population. To avoid sampling bias, we abstained from expanding our inclusion dates beyond September 30, 2015 to avoid any inconsistencies in diagnoses following the ICD-9 to ICD-10 transition (beginning October 1, 2015) as others have reported [25]. An additional strength of our study was the ability to review recurrent visits in detail to exclude follow-up visits and asymptomatic bacteriuria cases from true recurrences, adding confidence to our findings.

## CONCLUSIONS

In conclusion, longer treatment duration in outpatient settings was associated with increased recurrence of UTI in men without additional complicating factors. Our study adds evidence that men with UTI and no additional complicating factor such as prostatitis can be treated with a 7-day antibiotic course. Shorter duration of antibiotic treatment for male UTI may lead to decreased risk of antibiotic resistance, fewer adverse effects, and lower costs. We are currently conducting a randomized, controlled trial of 7 versus 14 days of antibiotics for male UTI that will bring further evidence to guide clinical practice [26, 27].
